# Ginsenoside Rg1 Ameliorates LPS‐Induced Sepsis‐Associated Lung Injury in Mice via VEGFC/D‐VEGFR3 Signaling‐Mediated Lymphangiogenesis and Lymphatic Remodeling

**DOI:** 10.1002/kjm2.70229

**Published:** 2026-05-07

**Authors:** He Wang, Lan Hu, Chun‐Pan Zhang, Wen‐Jie Qi, Yu‐Guo Song

**Affiliations:** ^1^ Department of Infection Beijing Friendship Hospital, Capital Medical University Beijing China; ^2^ Department of Occupational Medicine and Clinical Toxicology Beijing Chaoyang Hospital, Capital Medical University Beijing China

**Keywords:** ginsenoside Rg1, LPS, lymphangiogenesis, lymphatic clearance, sepsis

## Abstract

Sepsis‐induced acute lung injury (ALI) remains challenging to treat, with conventional anti‐inflammatory therapies offering limited efficacy. The lymphatic system is crucial for removing edema and inflammatory mediators, and its impairment can exacerbate lung injury. Ginsenoside Rg1, a bioactive botanical compound, has diverse pharmacological properties, but its ability to modulate lymphangiogenesis in septic ALI is unclear. This study investigated whether Ginsenoside Rg1 can alleviate lipopolysaccharide (LPS)‐induced ALI in mice by promoting lymphangiogenesis via the vascular endothelial growth factor C (VEGFC)/D‐VEGF receptor 3 (VEGFR3) signaling pathway. ALI was induced in C57BL/6 mice by intraperitoneal injection of LPS (10 mg/kg). Mice were treated with Rg1 at low (30 mg/kg) or high (60 mg/kg) doses. Lung injury was assessed by wet‐to‐dry weight ratios, vascular permeability, histopathology, and lymphatic vessel density. Levels of inflammatory cytokines, VEGFC/D, VEGFR3, and downstream signaling molecules were measured by enzyme‐linked immunosorbent assay, western blotting, and qPCR. Ginsenoside Rg1 treatment dose‐dependently reduced pulmonary edema, vascular leakage, and histological damage. It also reversed LPS‐induced decreases in VEGFC/D and increased VEGFR3 expression, resulting in enhanced lymphatic vessel density. Rg1 activated VEGFR3 downstream pathways (ERK/Prox‐1, AKT) and upregulated lymphatic function genes (*CCL21a, ACKR2*). Ginsenoside Rg1 can attenuate septic ALI by stimulating the VEGFC/D‐VEGFR3 axis to promote functional lymphangiogenesis, thereby facilitating clearance of edema and inflammatory mediators. This mechanism offers a novel therapeutic strategy focused on enhancing clearance rather than only on suppressing inflammation, potentially reducing tissue damage and the side effects associated with conventional treatments.

**Abbreviations**
ACKR2: Atypical Chemokine Receptor 2
AKT: Protein Kinase B
ALI: Acute Lung Injury
ARDS: Acute Respiratory Distress Syndrome
BALF: Bronchoalveolar lavage fluid
BCA: Bicinchoninic Acid
CCL21: C–C Motif Chemokine Ligand 21
DAPI: 4′,6‐Diamidino‐2‐Phenylindole
EB: Evans Blue
ELISA: Enzyme‐Linked Immunosorbent Assay
ERK: Extracellular Signal‐Regulated Kinase
GAPDH: Glyceraldehyde‐3‐Phosphate Dehydrogenase
HRP: Horseradish Peroxidase
IL: Interleukin
LIS: Lung Injury Score
LPS: Lipopolysaccharide
LYVE‐1: Lymphatic Vessel Endothelial Hyaluronan Receptor‐1
OCT: Optimal Cutting Temperature Compound
pAKT: Phosphorylated Protein Kinase B
PBS: Phosphate‐Buffered Saline
PCR: Polymerase Chain Reaction
pERK: Phosphorylated Extracellular Signal‐Regulated Kinase
PMSF: Phenylmethylsulfonyl Fluoride
Prox‐1: Prospero Homeobox Protein 1
qRT‐PCR: Quantitative Reverse Transcription Polymerase Chain Reaction
RIPA: Radioimmunoprecipitation Assay
RNA: Ribonucleic Acid
SD: Standard Deviation
SEM: Standard Error of the Mean
TNF‐α: Tumor Necrosis Factor‐alpha
VEGF: Vascular Endothelial Growth Factor
VEGFC/D: Vascular Endothelial Growth Factor C/D
VEGFR3: Vascular Endothelial Growth Factor Receptor 3
W/D Ratio: Wet‐to‐Dry Weight Ratio

## 1 Introduction

Management of acute lung injury (ALI) induced by sepsis continues to represent a significant challenge within intensive care settings. Existing therapeutic approaches primarily aim to mitigate vascular permeability and suppress systemic inflammatory responses. However, the role of the lymphatic system, which contributes to the clearance of interstitial fluid, immune cells, and metabolic waste, has been largely neglected in this context. Recent evidence suggests that disruption of lymphatic pathways may contribute to persistent pulmonary edema and delayed resolution of inflammation, thereby implicating a novel pathogenic mechanism in ALI [1]. Notably, multiple rigorous clinical trials have demonstrated that anti‐inflammatory treatments alone fail to reduce mortality in patients with acute respiratory distress syndrome (ARDS) and related conditions [2, 3, 4]. This ineffectiveness may be due to the immunosuppressive effects of interventions such as corticosteroids, which can compromise host defenses and increase susceptibility to secondary infections. These observations necessitate a paradigm shift in the therapeutic strategy: rather than only inhibiting inflammation, clearance of edema and inflammatory mediators may be more efficacious, based on the preservation of the protective functions of inflammation with minimization of its detrimental consequences.

This conceptual realignment is reminiscent of the ancient Chinese parable “Great Yu Controls the Flood,” wherein Great Yu managed flooding by creating drainage channels instead of erecting barriers. This analogy underscores the rationale for therapeutic approaches that prioritize the facilitation of resolution pathways over mere suppression.

At the molecular level, lymphangiogenesis is chiefly governed by the signaling axis between vascular endothelial growth factor C/D (VEGFC/D) and its receptor VEGFR3, which constitutes the primary and most evolutionarily conserved mechanism under both physiological and pathological conditions [5, 6]. While extensive research on lymphatic regeneration has been conducted in oncology, with debate persisting regarding its precise role [7, 8, 9], its function has been more clearly delineated in some chronic diseases, including cardiac repair [10] and occupational toxicosis [11]. Moreover, in the context of acute neurological disorders such as intracerebral hemorrhage and neuroinflammation, activation of this pathway has been shown to promote lymphangiogenesis and attenuate local inflammation [12, 13]. Nonetheless, the specific role and therapeutic potential of the VEGFC/D‐VEGFR3 axis in acute systemic inflammatory conditions, particularly sepsis‐induced ALI, remain inadequately characterized.

**FIGURE 1:**
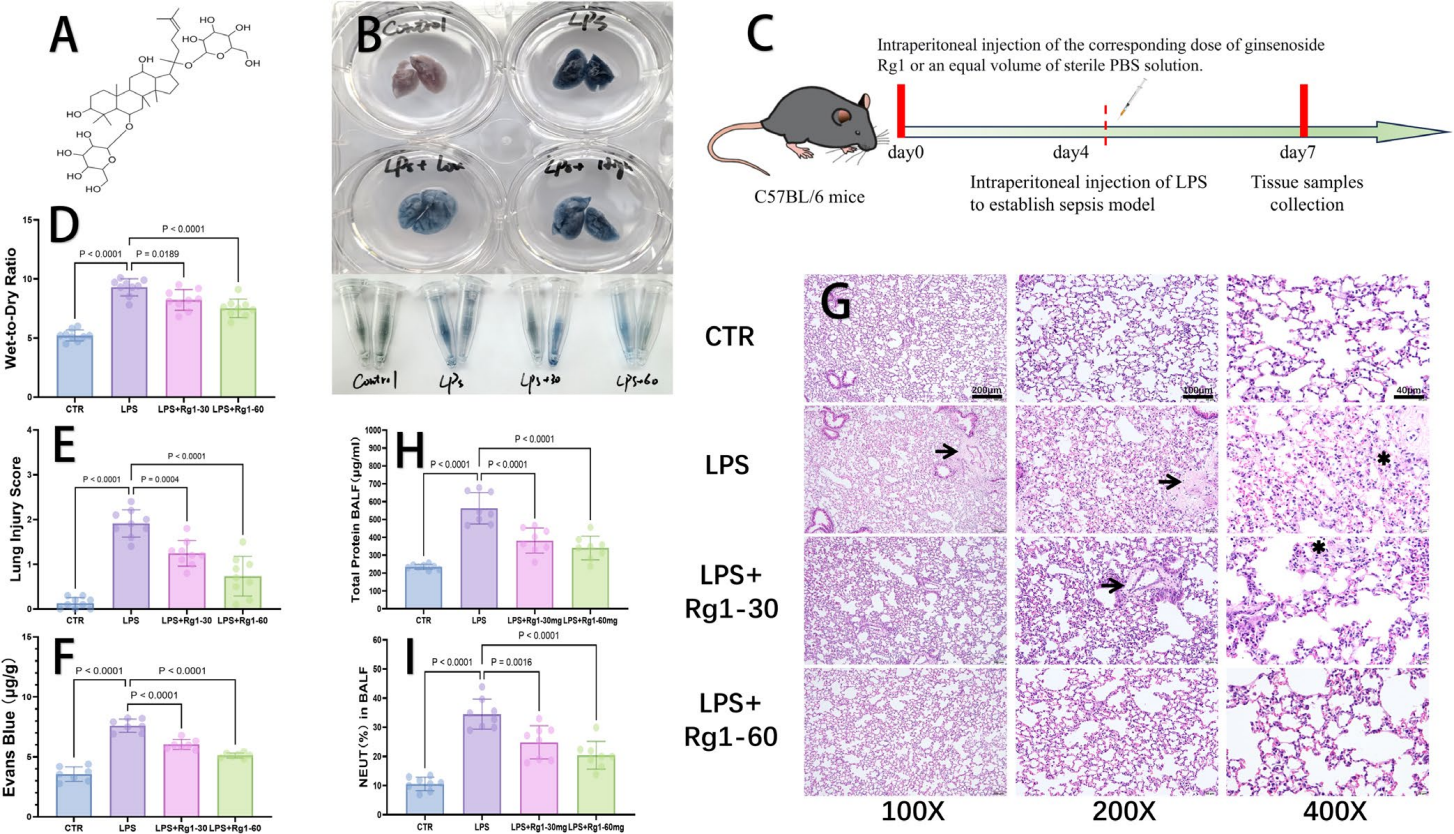
Protective effects of Ginsenoside Rg1 on LPS‐induced lung injury in septic mice. (A) Chemical structure of Ginsenoside Rg1 (Molecular formula: C₄₂H₇₂O₁₄; Molecular weight: 801.01). (B) Gross appearance of lung tissue and extracted EB dye solution from each group. The LPS group exhibited the most intense blue staining. (C) Schematic diagram of the experimental animal grouping and treatment timeline. (D) Lung wet‐to‐dry (W/D) weight ratio in each group. (E) Histopathological lung injury score (LIS) for each group. (F) Quantitative measurement of EB content (μg/g tissue) in lung homogenates. (G) Representative photomicrographs of H&E‐stained lung sections from each group (magnification: 100×, 200×, 400×; scale bars: 200 μm, 100 μm, 40 μm, respectively). Black arrows indicate abundant inflammatory exudates and neutrophil infiltration. The asterisk (*) denotes pronounced interstitial edema. High‐dose Ginsenoside Rg1 treatment markedly reduced pulmonary inflammatory infiltration and tissue edema in septic mice. (H) BALF total protein concentration for each group. (I) Neutrophil percentage in BALF for each group.

Ginsenoside Rg1 is a bioactive monomeric compound extracted from *Panax ginseng* (chemical structure depicted in Figure 1A) that possesses well‐documented anti‐inflammatory and pro‐angiogenic effects. Importantly, it has been shown to stimulate lymphangiogenesis in models of certain chronic and subacute diseases [14, 15]. However, its capacity to activate the VEGFC/D‐VEGFR3 signaling pathway and induce functional lymphangiogenesis within the hyper‐acute, highly inflammatory environment characteristic of sepsis‐induced ALI has yet to be investigated.

To address this research gap, the present study was designed to systematically examine whether Ginsenoside Rg1 can mitigate lipopolysaccharide (LPS)‐induced ALI in a mouse model of sepsis by enhancing functional lymphangiogenesis. This study tested the hypothesis that Rg1 confers its protective effects by modulating the VEGFC/D‐VEGFR3 signaling axis and the downstream ERK/prospero homeobox protein 1 (Prox‐1) and AKT pathways, thereby facilitating lymphatic remodeling within pulmonary tissue. This investigation aimed not only to elucidate a novel therapeutic mechanism of a natural compound but also to propose an innovative adjunctive “clearance‐enhancing” strategy for sepsis management, thereby providing an alternative therapeutic approach that promotes resolution in conjunction with conventional treatments.

## 2 Materials and Methods

### 2.1 Reagents

The Ginsenoside Rg1 utilized in this investigation was procured from Shanghai Yuan Ye Bio‐Technology Co. Ltd. (CAS number 22427‐39‐0; molecular formula C42H72O14; molecular weight 801.01; catalog number B21057). As illustrated in Figure 1A, the compound was dissolved in sterile, endotoxin‐free phosphate‐buffered saline (PBS) to prepare 3 and 6 mg/mL solutions, which were subsequently stored at −20°C for short‐term preservation. LPS derived from *Escherichia coli* O111:B4 was obtained from Beijing Kuerhui Technology Co. Ltd. (CAS number 93572‐42‐0; catalog number K08573) and prepared for use by dissolving in PBS to a concentration of 1 mg/mL, following an identical storage protocol.

### 2.2 Animals and Treatments

The experiments were conducted between October 2025 and December 2025. Male C57BL/6 mice, aged 8–10 weeks and weighing 20–25 g, were procured from the Experimental Animal Center of Capital Medical University (Beijing, China). All animals were maintained in a single, air‐conditioned environment with controlled temperature (20°C–22°C) and humidity (50%–70%), under a 12‐h light/dark cycle, with unrestricted access to food and water. Mice were used in experiments after a 1‐week acclimatization period. Of the 40 mice used in this study, 8 mice were used in preliminary pilot studies and 32 in the primary experiments. The mice were randomly assigned to four groups (*n* = 8 per group) to receive the following treatments: control (control group), LPS (LPS group), LPS plus low‐dose Ginsenoside Rg1 (30 mg/kg; low‐dose group), and LPS plus high‐dose Ginsenoside Rg1 (60 mg/kg; high‐dose group).

All experimental protocols adhered to the National Research Council's Guide for the Care and Use of Laboratory Animals (China), the ARRIVE guidelines for reporting in vivo research, and the European Union Directive 2010/63/EU concerning the protection of animals used for scientific purposes. Ethical approval was granted by the Animal Protection and Use Committee of Beijing Friendship Hospital, Capital Medical University (Approval No. 25‐2060).

Mice assigned to the low‐dose group received intraperitoneal injections of Ginsenoside Rg1 at 30 mg/kg twice daily, whereas the high‐dose group received injections of Ginsenoside Rg1 at 60 mg/kg on the same schedule. The control and LPS‐only groups were injected with equivalent volumes of sterile, endotoxin‐free PBS (0.01 M, pH 7.4). Pretreatment was conducted over 3 consecutive days to establish steady‐state drug concentrations. On the fourth day, following a 24‐h fasting period, mice in the LPS, low‐dose, and high‐dose groups were given a single intraperitoneal injection of LPS at 10 mg/kg; control animals received PBS. Administration of the drug or vehicle continued post‐challenge. At 72 h after LPS administration, the mice were euthanized under anesthesia induced by 3%–5% isoflurane. Blood samples were collected via ocular enucleation. Subsequently, bronchoalveolar lavage fluid (BALF) was collected, followed by transcardial perfusion. Lung and other relevant tissues were harvested for further analyses. The experimental timeline is depicted in Figure 1C.

The LPS dose of 10 mg/kg and a 72‐h observation period were chosen based on preliminary studies aimed at establishing a consistent and severe model of acute lung injury. This model facilitates the evaluation of therapeutic interventions and the mechanistic study of lymphangiogenesis, while avoiding excessive mortality that would hinder the assessment of resolution processes. Humane endpoints were rigorously monitored throughout the study. Mice displaying moribund conditions, such as inability to access food or water, pronounced lethargy, or prolonged immobility, were to be euthanized immediately to alleviate suffering. All animals survived until the 72‐h endpoint, at which time tissues were harvested for subsequent analyses. Notably, one mouse in the LPS‐treated group experienced cardiac arrest during anesthesia induction immediately prior to euthanasia; nevertheless, tissues were promptly collected, and data from this subject were included in all reported results.

### 2.3 Assessment of Pulmonary Vascular Permeability

Pulmonary microvascular permeability was assessed using the Evans Blue (EB) dye extravasation assay, conducted in accordance with a rigorously validated protocol previously optimized by our research team [11]. In brief, a 2% (w/v) EB dye solution (CAS 314‐13‐6; Catalog No.: E6135, Macklin, Shanghai, China) was prepared in saline. Mice were given an intravenous injection of the EB solution via the tail vein at a dosage of 4 mL per kilogram of body weight. After the dye was permitted to circulate for 2 h, with the mice under deep anesthesia, the systemic vasculature was perfused transcardially with ice‐cold PBS to remove intravascular dye. After the mice had been euthanized, the right anterior lobe of the lung was excised, weighed, and incubated in formamide (CAS 75‐12‐7; Catalog No.: F809511, Macklin) at a volume of 2 mL per gram of tissue for 24 h at 37°C to facilitate dye extraction. The concentration of EB in the formamide extract was quantified spectrophotometrically at 620 nm using a microplate reader (Thermo Fisher Scientific, USA). The EB content within the tissue was determined by reference to a standard curve of EB in formamide and expressed as micrograms of EB per gram of wet lung tissue, calculated using the formula: EB concentration (μg/g tissue) = [EB] in formamide (μg/mL) × volume of formamide (mL)/wet lung tissue weight (g).

### 2.4 Measurement of the Lung Wet‐To‐Dry Weight Ratio

Pulmonary edema, a well‐established indicator of ALI [1, 4], was quantitatively evaluated by measuring the lung wet‐to‐dry (W/D) weight ratio. At 72 h after LPS administration, the left lung lobe was excised, and its wet weight was immediately recorded. Subsequently, the tissue was wrapped in aluminum foil and dried in an oven at 80°C for 24 h until a constant dry weight was achieved. The W/D ratio was then calculated to assess the extent of pulmonary edema.

### 2.5 Histopathological Examination

For histopathological evaluation, the right middle lung lobe was fixed in 4% paraformaldehyde solution (CAS 30525‐89‐4; Catalog No.: BL539A, Biosharp, Beijing, China), embedded in paraffin, and sectioned into 5‐μm thick slices. These sections were mounted on glass slides and stained with hematoxylin and eosin (H&E) to examine morphological changes. The stained sections were observed and imaged using an upright light microscope (Olympus BX53F2, Evident, Japan). To maintain objectivity, a blinded pathologist assessed and scored lung injury using a standardized lung injury score (LIS).

### 2.6 Bronchoalveolar Lavage Fluid Analysis

The protein concentration in the supernatant was quantified utilizing a BCA Protein Assay Kit (Catalog No.: P0399; Beyotime, Shanghai, China) in accordance with the manufacturer's protocol. Subsequently, the cell pellet was resuspended in PBS, and both total and differential cell counts were assessed employing a fully automated hematology analyzer (XT‐2000i, Sysmex, Kobe, Japan).

### 2.7 Enzyme‐Linked Immunosorbent Assay (ELISA)

For measurement of serum cytokine and growth factor concentrations, mice were anesthetized with isoflurane, and the periocular region was disinfected with an alcohol swab. Terminal blood samples were collected via enucleation, yielding approximately 0.5–0.8 mL of whole blood per animal. Serum was separated by centrifugation at 2000–3000*g* for 15–20 min at 4°C, and the supernatant was carefully harvested for subsequent analysis.

Serum concentrations of VEGFC (Catalog No.: E03V0013), VEGFD (Catalog No.: E03V0012), interleukin 6 (IL‐6, Catalog No.: E03I0006), and tumor necrosis factor‐alpha (TNF‐α, Catalog No.: E03T0008) were determined using commercially available ELISA kits (all from BLUE GENE, Shanghai, China) in strict accordance with the manufacturer's protocols. All standards, controls, and samples were assayed in duplicate. Absorbance was measured at 450 nm using a microplate reader (Thermo Fisher Scientific). Concentrations were calculated by interpolating sample absorbance values against a standard curve generated through nonlinear regression analysis.

### 2.8 Immunofluorescence and Fluorescence Microscopy

Fresh tissue samples obtained from the right lower lobe of the lung were embedded in Optimal Cutting Temperature (OCT) compound (Catalog number: G6059‐110ML, Servicebio, Wuhan, China) and rapidly frozen by immersion in liquid nitrogen. Cryosections with a thickness of 5 μm were prepared, mounted onto slides, and gently rinsed twice with PBS. The sections were then fixed in 4% paraformaldehyde for 15 min at room temperature, followed by incubation in a blocking solution comprising 1% bovine serum albumin (BSA) and 10% normal goat serum for 1 h to minimize non‐specific antibody binding. The sections were then incubated overnight at 4°C with a primary antibody targeting VEGFR3 (dilution 1:300; Catalog number: ab27278, Abcam, Cambridge, UK). After thorough washing, the sections were incubated for 1 h at room temperature with an Alexa Fluor 594‐conjugated goat anti‐rabbit IgG (H + L) secondary antibody (dilution 1:500; Catalog number: ab150080, Abcam) to detect VEGFR3 expression, which was visualized as a red fluorescence signal. Nuclear counterstaining was performed using DAPI (blue fluorescence; Catalog number: ab104139, Abcam). Imaging and observation of the stained sections were conducted using an inverted fluorescence microscope (Olympus IX73). For quantitative assessment, 20 randomly selected microscopic fields were captured per group. Lymphatic vessel density, defined as the area exhibiting positive VEGFR3 staining, was quantified utilizing ImageJ software (National Institutes of Health, Bethesda, MD, USA).

### 2.9 Quantitative Reverse Transcription PCR (qRT‐PCR)

Total RNA was isolated from freshly harvested left lung tissue utilizing TRIzol reagent (Catalog No. 15596026CN, Thermo Fisher Scientific) in accordance with the manufacturer's protocol. Subsequent reverse transcription and quantitative real‐time PCR analyses were conducted as previously described [16]. The primer sequences employed for amplification are listed in Table 1. Quantitative PCR assays were carried out using SYBR Green qPCR Master Mix (Catalog No. A6015, Sigma‐Aldrich, St. Louis, MO, USA) on a Fast 7500 Real‐Time PCR System (Thermo Fisher Scientific), following the manufacturer's recommended thermal cycling conditions. Gene expression levels were normalized against the housekeeping gene *glyceraldehyde‐3‐phosphate dehydrogenase* (*GAPDH*). Relative expression was determined using the 2^−ΔΔCt^ method and expressed as fold change relative to the control group, which was assigned a value of 1.

**TABLE 1:** Primer sequences used for qRT‐PCR.

Gene	Forward Primer (5′ → 3′)	Reverse Primer (5′ → 3′)
*VEGFC*	GAGGTCAAGGCTTTTGAAGGC	CTGTCCTGGTATTGAGGGTGG
*VEGFD*	TTGAGCGATCATCCCGGTC	GCGTGAGTCCATACTGGCAAG
*VEGFR3*	CTGGCAAATGGTTACTCCATGA	ACAACCCGTGTGTCTTCACTG
*LYVE‐1*	CAGCACACTAGCCTGGTGTTA	CGCCCATGATTCTGCATGTAGA
*Prox‐1*	AGAAGGGTTGACATTGGAGTGA	TGCGTGTTGCACCACAGAATA
*CCL21a*	GTGATGGAGGGGGTCAGGA	GGGATGGGACAGCCTAAACT
*ACKR2*	TTTGCAGGAAGGACGAGGTC	CCCAGAAGGGCATAGTCACTAC
*GAPDH*	AGGTCGGTGTGAACGGATTTG	TGTAGACCATGTAGTTGAGGTCA

*Note:* All primer sequences are shown from 5′ to 3′.

Abbreviations: ACKR2, atypical chemokine receptor 2; CCL21a, chemokine (C–C motif) ligand 21a; GAPDH, glyceraldehyde‐3‐phosphate dehydrogenase (used as an internal control); LYVE‐1, lymphatic vessel endothelial hyaluronan receptor 1; Prox‐1, prospero homeobox protein 1; VEGFC, vascular endothelial growth factor C; VEGFD, vascular endothelial growth factor D; VEGFR3, vascular endothelial growth factor receptor 3.

### 2.10 Western Blot Analysis

Lung tissue obtained from the left lobe was homogenized and lysed using radioimmunoprecipitation assay (RIPA) lysis buffer (Catalog number: HX60127; Huaxingbio, Beijing, China) supplemented with 1 mM phenylmethylsulfonyl fluoride (PMSF), a protease inhibitor cocktail, and a phosphatase inhibitor cocktail. The homogenate was subjected to centrifugation at 15,000*g* for 15 min at 4°C. The resulting supernatant was collected, and the total protein concentration was measured utilizing a bicinchoninic acid (BCA) protein assay kit (Catalog number: HX18651; Huaxingbio, Beijing, China) in accordance with the manufacturer's instructions. Protein samples (20 μg per lane) were resolved by electrophoresis on 8% or 12% sodium dodecyl sulfate‐polyacrylamide gel electrophoresis (SDS‐PAGE) gels (Catalog No.: E302‐01 and E304‐01; Vazyme, Nanjing, China) alongside a prestained protein ladder (PageRuler, Catalog No.: 26616; Fermentas, MA, USA). After electrophoresis, proteins were transferred onto polyvinylidene difluoride (PVDF) membranes (Immobilon‐P; Millipore, MA, USA) via electroblotting. Membranes were blocked with Rapid Blocking Buffer (Catalog No.: P30500; NCM Biotech, Suzhou, China) for 1 h at room temperature, followed by overnight incubation at 4°C with primary antibodies targeting β‐actin (1:1000; Catalog No.: ab8227; Abcam), β‐tubulin (1:1000; Catalog No.: 10094‐1‐AP; Proteintech, USA), VEGFR3 (1:1000; Catalog No.: ab300403; Abcam), VEGFC (1:1000; Catalog No.: A12530; Abclonal, Wuhan, China), VEGFD (1:1000; Catalog No.: A19242; Abclonal), ERK (1:1000; Catalog No.: ab184699; Abcam), phosphorylated ERK (pERK) (1:1000; Catalog No.: ab278538; Abcam), AKT (1:1000; Catalog No.: 4685; CST, MA, USA), phosphorylated AKT (pAKT) (1:1000; Catalog No.: ab81283; Abcam), Prox‐1 (1:1000; Catalog No.: A9047; Abclonal), and LYVE‐1 (1:1000; Catalog No.: ab218535; Abcam).

Given the potential overlap between the molecular weights of certain target proteins and loading controls, two distinct loading controls, β‐actin and β‐tubulin, were employed. For the detection of proteins and their phosphorylated forms, membranes underwent a stripping and re‐probing procedure. Specifically, following initial signal detection, membranes were treated with Antibody Stripping Solution (Catalog No.: G2079‐100ML; Servicebio, Wuhan, China) according to the manufacturer's protocol, re‐blocked, and subsequently incubated with the next primary antibody.

After primary antibody incubation, membranes were washed and incubated with a horseradish peroxidase (HRP)‐conjugated goat anti‐rabbit IgG secondary antibody (1:2000; Catalog No.: ab205718; Abcam) for 1 h at room temperature. Immunoreactive bands were visualized using a highly sensitive enhanced chemiluminescence (ECL) substrate kit (Super ECL Plus; Catalog No.: P1050; PuliLai, Beijing, China) and imaged with a chemiluminescence detection system. Band intensities were quantified by densitometric analysis using ImageJ software. Protein expression levels were determined based on data obtained from a minimum of three independent experiments.

### 2.11 Statistical Analysis

Statistical analyses were conducted utilizing GraphPad Prism (version 10.4.0) and SPSS (version 19.0) software. Data are expressed as mean ± standard deviation (SD) or mean ± standard error of the mean (SEM), as specified. Statistical significance was defined by a *p*‐value less than 0.05. All experiments were independently replicated at least three times. Following verification of data normality, one‐way analysis of variance (ANOVA) was employed for multiple group comparisons. In cases where assumptions of normality or homogeneity of variance were not met, non‐parametric methods were applied, specifically the Kruskal–Wallis test for multiple comparisons, followed by Dunn's post hoc test. For comparisons between two groups, the unpaired two‐tailed Student's *t*‐test was utilized. A threshold of *p* < 0.05 was applied to determine statistical significance.

## 3 Results

### 3.1 Ginsenoside Rg1 Treatment Ameliorates LPS‐Induced Lung Injury in Septic Mice

All 32 mice (*n* = 8 per group) survived until the 72‐h time point following LPS administration and were incorporated into the final analyses. Among the four experimental groups, the LPS‐treated mice demonstrated the most severe overall condition; however, no mortality was recorded throughout the 72‐h observation period. Notably, one mouse in the LPS group experienced cardiac arrest during anesthesia induction immediately prior to euthanasia; nevertheless, tissue samples were promptly harvested, and the corresponding data were included in all analyses presented herein. No deaths or anesthesia‐related adverse events were observed in the Control, LPS + Rg1 low‐dose, or LPS + Rg1 high‐dose groups. The ALI model was effectively established in all LPS‐challenged mice, as evidenced by significant increases in pulmonary edema, vascular permeability, histopathological alterations, and inflammatory cytokine expression, as detailed below.

Administration of Ginsenoside Rg1 significantly and dose‐dependently mitigated LPS‐induced pulmonary edema in mice with sepsis. The lung wet‐to‐dry weight ratio was substantially increased in LPS‐treated mice (9.29 ± 0.72) relative to that in the control group (5.21 ± 0.47; *p* < 0.0001). This elevation was not observed in mice treated with low‐dose Rg1 (8.22 ± 0.87; *p* = 0.0189 versus LPS group), and the lung wet‐to‐dry weight ratio was even less in mice treated with high‐dose Rg1 (7.51 ± 0.77; *p* < 0.0001 versus LPS group; Figure 1D).

Consistent with the reduction in edema, treatment with Ginsenoside Rg1 also attenuated pulmonary vascular permeability. The EB extravasation assay results demonstrated that LPS challenge significantly increased vascular leakage, as evidenced by an increase in lung EB content from 3.56 ± 0.60 μg/g in the control group to 7.60 ± 0.55 μg/g in the LPS group (*p* < 0.0001; Figure 1B,F). Treatment with Rg1 dose‐dependently reduced this LPS‐induced vascular permeability, lowering the EB content to 6.03 ± 0.41 μg/g in the low‐dose group and further to 5.12 ± 0.21 μg/g in the high‐dose group (both *p* < 0.0001 versus LPS group).

The total protein concentration in BALF was significantly elevated in mice subjected to LPS challenge (563.13 ± 88.09 μg/mL) relative to the control group (234.61 ± 14.15 μg/mL, *p* < 0.0001). Administration of Ginsenoside Rg1 resulted in a dose‐dependent attenuation of this increase, with total protein levels measured at 381.81 ± 70.54 μg/mL in the low‐dose cohort and 340.40 ± 66.31 μg/mL in the high‐dose cohort, both values significantly reduced compared to the LPS group (*p* < 0.0001). Correspondingly, the proportion of neutrophils in BALF was markedly elevated following LPS exposure (34.36% ± 5.18%) compared to controls (10.56% ± 2.28%, *p* < 0.0001). Treatment with Rg1 dose‐dependently mitigated neutrophilic infiltration, decreasing the neutrophil percentage to 24.86% ± 5.69% in the low‐dose group (*p* = 0.0016 versus LPS) and further to 20.40% ± 4.75% in the high‐dose group (*p* < 0.0001 versus LPS).

Histopathological analysis of H&E‐stained lung sections corroborated the protective effects of Rg1. LPS exposure resulted in pronounced pulmonary injury, characterized by alveolar collapse, interstitial thickening, marked inflammatory cell infiltration, and intra‐alveolar exudation (Figure 1G). This damage was quantitatively reflected by a significantly greater LIS in the LPS group (1.91 ± 0.31) than in the control group (0.13 ± 0.12; *p* < 0.0001). Administration of Ginsenoside Rg1 ameliorated these pathological changes in a dose‐dependent manner, as indicated by significantly lower LIS values of 1.24 ± 0.29 in the low‐dose group (*p* = 0.0004 versus LPS group) and 0.73 ± 0.44 in the high‐dose group (*p* < 0.0001 versus LPS group; Figure 1E).

### 3.2 Ginsenoside Rg1 Upregulates VEGFR3 Expression and Induces Lymphangiogenesis in the Lungs of Septic Mice

**FIGURE 2:**
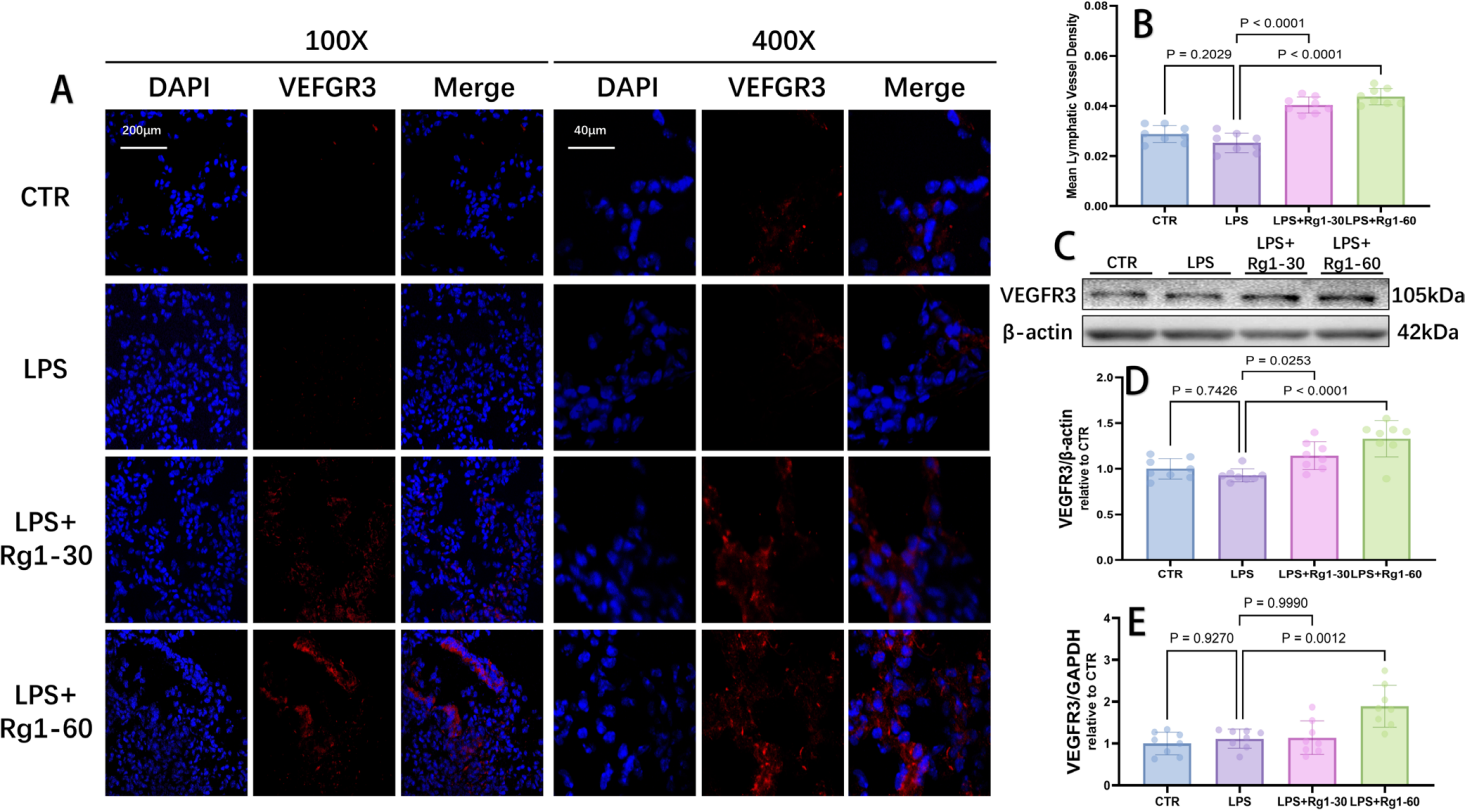
Ginsenoside Rg1 promotes VEGFR3 expression and increases lymphatic vessel density in the lungs of septic mice. (A) Immunofluorescence staining showing VEGFR3‐positive lymphatic structures (red, VEGFR3; blue, DAPI nuclear stain) in sub‐pleural lung tissue observed under a confocal microscope (magnification: 100×, 400×; scale bars: 200 μm, 40 μm). (B) Quantitative analysis of lymphatic vessel density in lung tissues. (C) Representative western blot bands showing VEGFR3 and the loading control β‐actin protein expression in lung tissues. (D) Relative quantitative analysis of VEGFR3 protein expression normalized to β‐actin. (E) Relative mRNA expression levels of *VEGFR3* in lung tissues measured by qRT‐PCR and normalized to *GAPDH* expression.

Immunofluorescence staining of VEGFR3, a well‐established marker of lymphatic endothelial cells [5], was employed to evaluate pulmonary lymphangiogenesis. Quantitative assessment indicated a non‐significant trend toward reduced sub‐pleural lymphatic vessel density in the LPS‐treated group compared with the control group (*p* = 0.2029). Conversely, administration of Ginsenoside Rg1 elicited a pronounced, dose‐dependent increase in VEGFR3‐positive lymphatic structures (Figure 2A). Quantitative measurements confirmed a significant increase in lymphatic vessel density in Rg1‐treated groups relative to both the control and LPS groups (Figure 2B). This morphological enhancement was confirmed at the molecular level. Western blot analysis of lung tissue homogenates revealed dose‐dependent upregulation of VEGFR3 protein expression following Rg1 treatment. Specifically, VEGFR3 expression in the LPS group (0.93 ± 0.07 relative to β‐actin) did not differ significantly from that in the control group (1.00 ± 0.11; *p* = 0.7426), whereas both the low‐dose (1.14 ± 0.15; *p* = 0.0253 vs. LPS) and high‐dose (1.33 ± 0.20; *p* < 0.0001 vs. LPS) groups exhibited significantly increased VEGFR3 expression (Figure 2C,D). Complementary qRT‐PCR analysis demonstrated parallel upregulation of *VEGFR3* mRNA expression in lung tissue, with the most substantial increase observed in the high‐dose group (1.89 ± 0.50‐fold change relative to control), in which *VEGFR3* expression significantly exceeded that in the LPS group (1.11 ± 0.23; *p* = 0.0012; Figure 2E). Collectively, these findings suggest that Ginsenoside Rg1 promotes both the expression and functional expansion of the lymphatic vasculature in injured pulmonary tissue, predominantly via upregulation of VEGFR3.

### 3.3 Ginsenoside Rg1 Alleviates Systemic Inflammation and Upregulates VEGFC/D Expression Both Systemically and Locally

**FIGURE 3:**
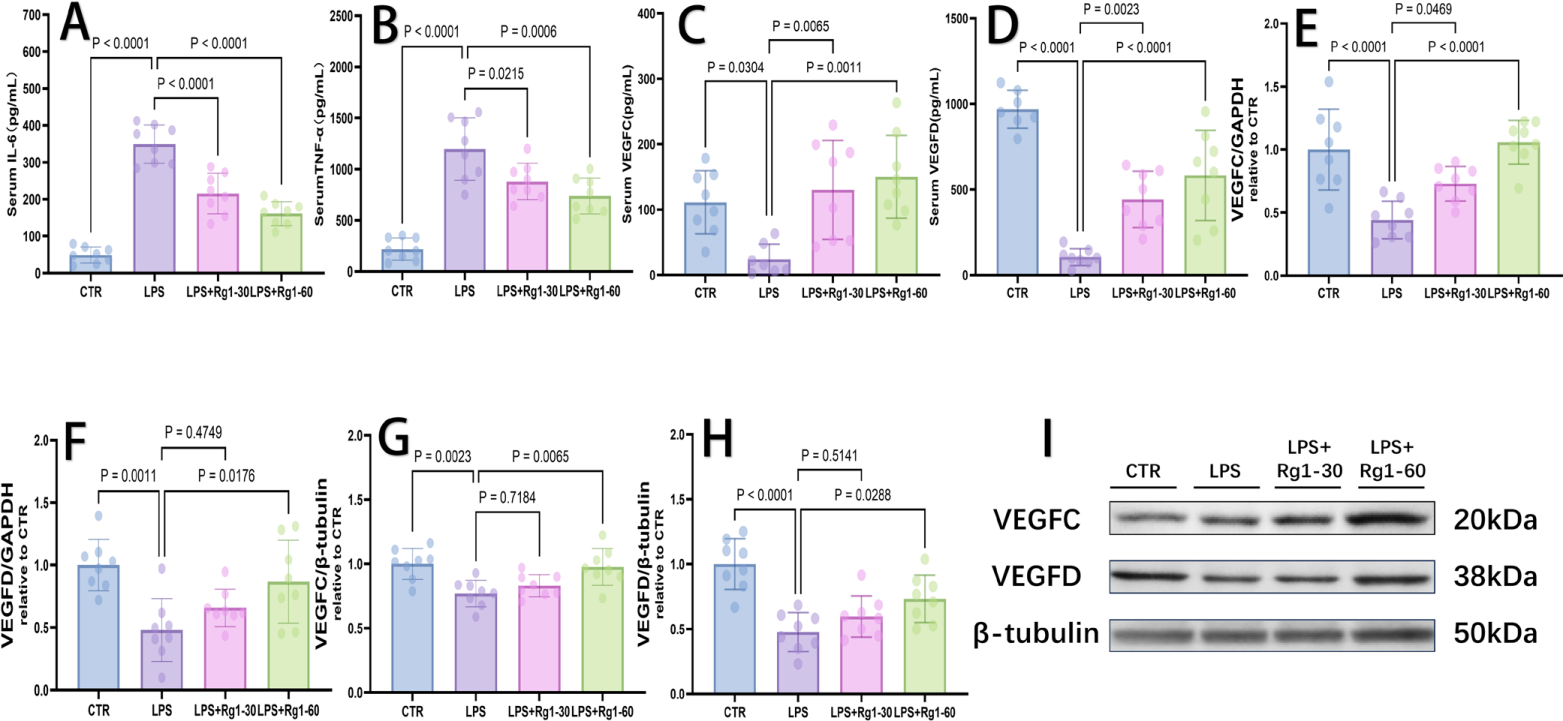
Ginsenoside Rg1 attenuates systemic inflammation and upregulates VEGFC/D expression both systemically and locally. (A) Serum concentrations of IL‐6 (pg/mL). (B) Serum concentrations of TNF‐α (pg/mL). (C) Serum concentrations of VEGFC (pg/mL). (D) Serum concentrations of VEGFD (pg/mL). (E) Relative mRNA expression levels of *VEGFC* in lung tissues measured by qRT‐PCR. (F) Relative mRNA expression levels of *VEGFD* in lung tissues measured by qRT‐PCR. (G) Relative quantitative analysis of VEGFC protein expression in lung tissues by western blotting, normalized to β‐tubulin expression. (H) Relative quantitative analysis of VEGFD protein expression in lung tissues by western blotting, normalized to β‐tubulin expression. (I) Representative western blot bands showing VEGFC, VEGFD, and the loading control β‐tubulin from lung tissues.

Subsequently, we investigated the effects of Ginsenoside Rg1 on critical lymphangiogenic mediators, specifically VEGFC and VEGFD, as well as its systemic anti‐inflammatory properties. At the systemic level, administration of Rg1 significantly alleviated the cytokine storm induced by LPS. Serum concentrations of pro‐inflammatory cytokines IL‐6 and TNF‐α were substantially higher in the LPS‐treated group (349.0 ± 51.91 pg/mL and 1197.0 ± 303.9 pg/mL, respectively) than in the control group (48.90 ± 21.73 pg/mL, *p* < 0.0001; 218.3 ± 108.5 pg/mL, *p* < 0.0001). Treatment with both low and high doses of Rg1 produced significant, dose‐dependent reductions in these cytokines (Figure 3A,B). Concurrently, Rg1 counteracted the LPS‐induced suppression of circulating lymphangiogenic factors. Specifically, the serum VEGFC level, which was markedly reduced after LPS exposure (23.81 ± 23.28 pg/mL versus 111.1 ± 48.43 pg/mL in control group; *p* = 0.0304), was restored to near‐control levels by both low‐dose and high‐dose Rg1 administration (130.0 ± 75.56 pg/mL and 150.2 ± 63.41 pg/mL in the low‐ and high‐dose groups, respectively; *p* = 0.0065 and *p* = 0.0011 compared to LPS; Figure 3C). A parallel trend was observed for serum VEGFD, with LPS inducing a significant reduction (105.6 ± 49.36 pg/mL versus 969.2 ± 111.0 pg/mL in controls; *p* < 0.0001) that was significantly ameliorated by Rg1 in a dose‐dependent manner (442.6 ± 164.3 pg/mL and 582.5 ± 263.3 pg/mL; *p* = 0.0023 and *p* < 0.0001 versus LPS; Figure 3D).

At the tissue level, quantitative PCR and western blot analyses of lung specimens confirmed that Rg1 treatment directly enhanced VEGFC/D signaling within the affected organ. LPS challenge resulted in significant downregulation of *VEGFC* and *VEGFD* mRNA expression in lung tissue compared with that in the control group (0.44 ± 0.15 versus 1.00 ± 0.32, *p* < 0.0001; 0.48 ± 0.25 versus 1.00 ± 0.21, *p* = 0.0011). However, Rg1 treatment dose‐dependently restored these transcript levels, with the high‐dose group exhibiting significant recovery (Figure 3E,F). This transcriptional upregulation was confirmed at the protein level, with western blot analysis demonstrating that the LPS‐induced decreases in pulmonary VEGFC and VEGFD protein expression were significantly reversed after high‐dose Rg1 treatment (Figure 3G–I).

Collectively, these findings indicate that Ginsenoside Rg1 effectively attenuates systemic inflammatory responses while concurrently restoring the expression of key lymphangiogenic factors VEGFC and VEGFD, both in the circulation and within the injured lung tissue.

### 3.4 Ginsenoside Rg1 Drives Lymphangiogenesis Through VEGFC/D‐VEGFR3‐Mediated ERK and AKT Activation, and Newly Formed Lymphatics Have Potential Pro‐Resolving Functions

To elucidate the downstream signaling mechanisms and functional capabilities of Rg1‐induced lymphangiogenesis, we investigated critical signaling pathways and gene expression markers.

#### 3.4.1 Activation of VEGFC/D‐VEGFR3 Signaling Pathways

**FIGURE 4:**
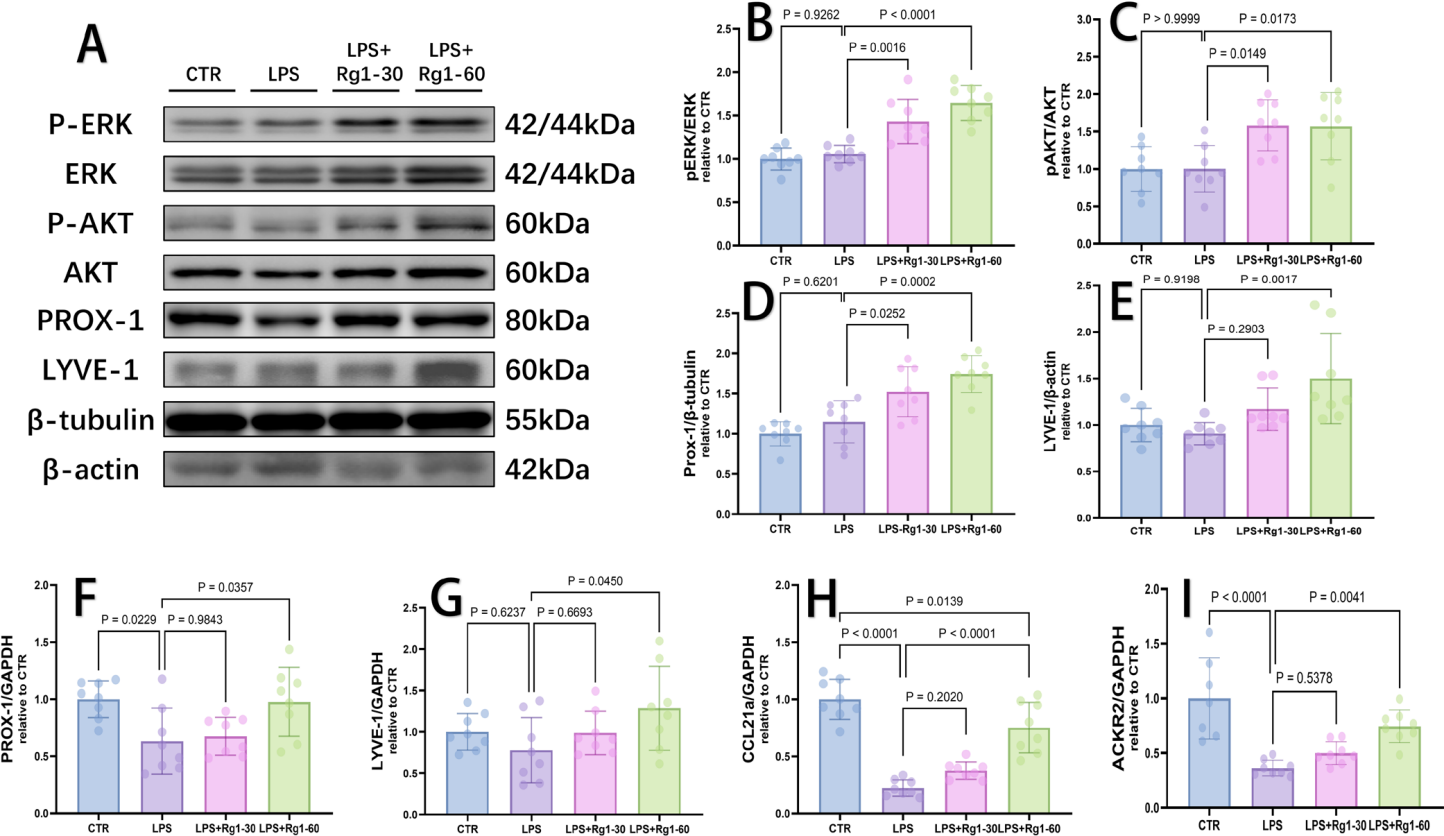
Ginsenoside Rg1 drives lymphangiogenesis by activating the VEGFC/D‐VEGFR3‐ERK‐Prox‐1 axis, co‐activates the AKT pathway to maintain lymphatic endothelial cell homeostasis, and upregulates lymphatic function‐related genes. (A) Representative western blot bands showing the expression of phosphorylated (p)‐ERK, total ERK, p‐AKT, total AKT, LYVE‐1, and Prox‐1 proteins in lung tissues. (B) Relative quantitative analysis of the p‐ERK/ERK protein ratio. (C) Relative quantitative analysis of the p‐AKT/AKT protein ratio. (D) Relative quantitative analysis of Prox‐1 protein expression normalized to β‐actin expression. (E) Relative quantitative analysis of LYVE‐1 protein expression normalized to β‐actin expression. (F) Relative mRNA expression levels of *Prox‐1* in lung tissues measured by qRT‐PCR and normalized to *GAPDH*. (G) Relative mRNA expression levels of *LYVE‐1* in lung tissues measured by qRT‐PCR and normalized to *GAPDH*. (H) Relative mRNA expression levels of *CCL21a* in lung tissues measured by qRT‐PCR. (I) Relative mRNA expression levels of *ACKR2* in lung tissues measured by qRT‐PCR.

Western blot analyses demonstrated that treatment with Rg1 significantly increased the phosphorylation levels of ERK and AKT, which are principal downstream effectors of VEGFR3 (Figure 4A). The ratio of phosphorylated ERK to total ERK (pERK/ERK) was significantly elevated in both the low‐dose (1.43 ± 0.26, *p* = 0.0016) and high‐dose (1.65 ± 0.20, *p* < 0.0001) groups compared with the LPS group (1.06 ± 0.10), which showed no significant difference from the control group (1.00 ± 0.13; *p* = 0.9262) (Figure 4B). Similarly, the phosphorylated AKT to total AKT ratio (pAKT/AKT) was significantly increased following Rg1 administration at both low (1.58 ± 0.34, *p* = 0.0149) and high doses (1.57 ± 0.45, *p* = 0.0173) compared with that in the LPS group (Figure 4C).

#### 3.4.2 Upregulation of Markers of Lymphatic Lineage and Maturation

The observed activation of the ERK and AKT signaling pathways correlated with the modulation of key transcriptional regulators governing lymphatic identity. Quantitative PCR analysis revealed that LPS exposure significantly suppressed *Prox‐1* mRNA expression (0.63 ± 0.29 versus 1.00 ± 0.16 in control group; *p* = 0.0229), and this effect was reversed by high‐dose Rg1 treatment (0.98 ± 0.30; *p* = 0.0357 versus LPS; Figure 4F). Correspondingly, Prox‐1 protein expression increased in a dose‐dependent manner with Rg1 administration (Figure 4D). *LYVE‐1* mRNA expression exhibited a similar pattern, with high‐dose Rg1 significantly augmenting its expression relative to that in the LPS group (1.29 ± 0.51 versus 0.78 ± 0.39; *p* = 0.0450; Figure 4G). Consistently, LYVE‐1 protein levels also demonstrated a dose‐dependent increase upon Rg1 treatment (Figure 4E).

#### 3.4.3 Potential Immunomodulatory Function of Newly Formed Lymphatic Vessels

To assess the immunological functionality of the induced lymphatics, we evaluated the expression of genes implicated in lymphatic transport and immune regulation. Quantitative PCR results indicated that LPS exposure resulted in downregulated expression of the mRNAs encoding chemokine (C–C motif) ligand 21a (CCL21a), a chemokine essential for immune cell trafficking, and atypical chemokine receptor 2 (ACKR2), a scavenger receptor involved in the degradation of inflammatory chemokines [17, 18, 19]. Notably, Rg1 treatment, particularly at the high dose, significantly restored the expression levels of *CCL21a* (0.75 ± 0.22 versus 0.22 ± 0.07 in the LPS group; *p* < 0.0001) and *ACKR2* (0.74 ± 0.15 versus 0.36 ± 0.07 in the LPS group; *p* = 0.0041) (Figure 4H,I). These findings suggest that lymphatics formed in response to Rg1 treatment may enhance immunomodulation, potentially contributing to the resolution of inflammation.

## 4 Discussion

The results of the present study provide evidence that pharmacological enhancement of lymphatic clearance offers a novel and feasible adjunctive approach for alleviating sepsis‐induced ALI. The proposed “drainage‐over‐suppression” paradigm, achieved by the natural compound Ginsenoside Rg1, aims to complement existing anti‐inflammatory treatments by facilitating functional lymphangiogenesis through activation of the VEGFC/D‐VEGFR3 signaling pathway. This alternative therapeutic strategy addresses a significant unmet clinical need, as sepsis‐induced ALI remains a leading cause of mortality in intensive care settings [20]. While infection control is essential, improving patient outcomes also requires mitigation of lung injury by promoting the clearance of alveolar fluid and inflammatory mediators [4, 21]. Notably, rigorous clinical trials have consistently demonstrated that administration of anti‐inflammatory interventions alone does not substantially reduce mortality rates [2, 3], highlighting the necessity for novel approaches. Emerging evidence implicating lymphatic dysfunction in the persistence of pulmonary edema has positioned the lymphatic system as a critical target for enhancing resolution of lung injury [1].

Accordingly, we hypothesized that pharmacological augmentation of lymphatic function could support the resolution of pulmonary edema and inflammation. In clinical practice in China, *Panax ginseng* is commonly incorporated in traditional medicinal formulations for sepsis management, including Shenfu injection, Qishen Huoxue Granule, and Shengmai injection. However, the complex and variable composition of the whole herb complicates controlled mechanistic investigations. Therefore, our focus was directed toward ginsenosides, the principal bioactive constituents of ginseng. While previous research from our institution identified at least 22 distinct types in Shenfu injection alone [22], we selected the monomeric compound Ginsenoside Rg1 based on its documented anti‐inflammatory [23], pro‐angiogenic [24], and pro‐lymphangiogenic effects in other disease models [14].

Our current findings demonstrate that Ginsenoside Rg1 significantly attenuated LPS‐induced lung injury in septic mice, and that this protective effect was closely linked to the promotion of pulmonary lymphangiogenesis. Mechanistically, Rg1 upregulated VEGFC/D and VEGFR3 expression both systemically and locally within the lung, thereby activating downstream ERK and AKT signaling pathways. This activation stimulated lymphatic endothelial cell proliferation and migration, resulting in remodeling of the lymphatic network and subsequent reduction of pulmonary edema. The protective properties of Ginsenoside Rg1 were further corroborated through analysis of BALF. Administration of Rg1 resulted in a dose‐dependent decrease in LPS‐induced total protein leakage and neutrophil infiltration within the BALF, suggesting a mitigation of alveolar‐capillary barrier disruption and pulmonary inflammation. These results are consistent with observed reductions in pulmonary edema, vascular permeability, and histopathological damage, collectively substantiating the therapeutic potential of Rg1 in the treatment of septic ALI. Furthermore, the observed upregulation of lymphatic function‐related genes, such as *CCL21* and *ACKR2*, indicates that the newly formed lymphatic vessels possessed the capacity to clear inflammatory mediators. Collectively, these results offer novel insights into the pathophysiology of septic ALI and provide compelling preclinical evidence supporting a therapeutic strategy focused on enhancing tissue fluid drainage rather than only suppressing inflammation. This clearance‐promoting approach respects the essential immune processes triggered by infection and, by avoiding broad immunosuppression, may reduce the risk of secondary infections.

Historically, the management of sepsis has concentrated on controlling infection and mitigating the excessive inflammatory response. Although these strategies are essential, they often produce suboptimal outcomes, analogous to the “blocking” approach described in the ancient Chinese parable “Great Yu Controls the Flood.” Drawing inspiration from the concept of “dredging” rather than “blocking,” the present study redirects therapeutic attention toward the lymphatic system, which plays a critical role in the clearance of excess tissue fluid, immune cells, and inflammatory mediators. In the LPS‐induced sepsis model, we observed pronounced pulmonary edema and excessive release of inflammatory mediators during the acute phase of sepsis. Utilizing the EB extravasation assay, which exploits the dye's albumin‐binding properties and the active drainage capacity of living cells to evaluate vascular permeability [11], we observed intense blue staining in lungs injured by LPS, indicative of increased cellular death and exudation. Treatment with Rg1 markedly attenuated this staining. This improvement was further supported by a reduction in the lung wet‐to‐dry weight ratio as well as histopathological observations, which revealed diminished interstitial edema, alveolar collapse, and neutrophilic infiltration after Rg1 administration.

To elucidate the mechanisms underlying the resolution of edema and inflammation achieved with Rg1 treatment, we examined lymphatic signaling pathways. LPS challenge significantly suppressed the levels of VEGFC/D in both plasma and lung tissue. Conversely, VEGFR3, a membrane‐bound receptor [25], was undetectable in plasma and exhibited only a non‐significant decreasing trend in lung tissue, potentially reflecting compensatory mechanisms aimed at preserving lymphatic function in response to injury. Notably, Rg1 treatment dose‐dependently restored the expression of these signaling molecules. Immunofluorescence microscopy confirmed that Rg1 significantly upregulated VEGFR3 expression concomitant with the amelioration of lung injury, consistent with findings from another interventional study [26]. Interestingly, while treatment with high‐dose Rg1 led to increased pulmonary VEGFR3 expression beyond the control level, local VEGFC/D protein expression in the high‐dose group did not exceed that in the control group. This apparent discrepancy may be attributed to the transcardial perfusion procedure employed, which likely removed blood‐derived proteins, including systemic VEGFC/D. The substantial restoration of plasma VEGFC/D concentrations after Rg1 treatment, combined with the production of VEGFC/D by known cellular sources such as platelets [27], fibroblasts [28], and macrophages [29], suggests that pulmonary lymphangiogenesis in this model is driven by both local and systemic factors, with systemic contributions potentially playing a more prominent role during the acute phase of sepsis‐induced ALI.

A principal finding of the present study is the marked reduction in VEGFC and VEGFD levels within the plasma and pulmonary tissues of mice subjected to LPS challenge, a result that contrasts with certain observations reported in oncological investigations. For example, Zhu et al. demonstrated that LPS treatment can enhance VEGFC expression in colorectal cancer cells, thereby facilitating lymphatic metastasis [30]. We hypothesize that this fundamental divergence arises from the distinct pathophysiological contexts involved. In the tumor microenvironment, LPS may serve as a persistent local stimulus that cancer cells exploit through receptors such as Toll‐like receptor 4 (TLR4) to activate pro‐metastatic signaling pathways, leading to the upregulation of VEGFC and subsequent co‐option of the lymphatic system to promote metastasis. Conversely, our model of severe systemic sepsis induces a life‐threatening cytokine storm, which involves substantial depletion, consumption, or functional impairment of VEGFC sources including platelets and M2 macrophages [31, 32], resulting in a pronounced decrease in VEGFC/D secretion. Consequently, the protective efficacy of Ginsenoside Rg1 is partially derived from its capacity to counteract this pathological suppression and restore the functionality of the VEGFC/D‐VEGFR3 signaling axis. This therapeutic objective contrasts with cancer treatment strategies aimed at inhibiting this pathway, underscoring the critical importance of tailoring interventions to specific disease contexts.

Consistent with previous findings [33], our results were further substantiated by evidence that Rg1 stimulation enhances the VEGFC/D‐VEGFR3 axis, thereby promoting ERK pathway‐mediated phosphorylation of VEGFR3 on endothelial cells. This phosphorylation event upregulates the master transcription factor Prox‐1, and the increased expression of Prox‐1 subsequently induces LYVE‐1 expression, culminating in lymphatic endothelial cell proliferation. These findings align with those of most prior studies [34, 35]. Moreover, as reported previously [36], the AKT signaling pathway plays a vital role in lymphatic endothelial cell migration, with AKT phosphorylation being more robustly activated by VEGFC than by VEGFA. Our data corroborate this, demonstrating that the AKT pathway is similarly activated and potentiated during Rg1‐mediated mitigation of LPS‐induced lung injury. Upon consideration of these findings with the existing literature, we conclude that the natural compound Ginsenoside Rg1 facilitates lymphatic endothelial cell proliferation and migration via canonical signaling pathways, thereby promoting structural regeneration and remodeling of the lymphatic vasculature.

Furthermore, qPCR analysis revealed a potential improvement in lymphatic function, involving CCL21a, a secreted factor, and ACKR2, a critical receptor, within lymphatic endothelial cells. CCL21 functions as a chemotactic signal by binding to and activating specific receptors on immune cells such as naïve T cells and dendritic cells, thereby directing their migration to targeted locations and facilitating localized immune surveillance [37]. Conversely, ACKR2 functions as a scavenger receptor with high affinity for various inflammatory chemokines, including CCL2 and CCL3. Rather than initiating conventional activation signaling, ACKR2 internalizes these chemokines and targets them for lysosomal degradation, contributing to the resolution of inflammatory signals and the regulation of excessive or chronic inflammation [37]. The observed expression of these genes subsequent to lymphangiogenesis implies that the newly formed lymphatic endothelium may play a role in modulating local inflammatory responses. It is noteworthy, however, that the expression levels of these functional genes, even after high‐dose Rg1 treatment, did not fully match those observed in the control group. This discrepancy, despite the marked increase in lymphatic vessel density detected via immunofluorescence, suggests that the functional maturation of the nascent lymphatic vessels remains incomplete, and that the new endothelial cells may require additional time to attain full functional competency.

It is important to highlight that the administration of a 10 mg/kg dose of LPS in this study induced severe pulmonary injury without causing mortality within a 72‐h period. This particular model was deliberately chosen to examine the therapeutic effects of Rg1 on lymphatic remodeling during the initial phases of ALI, as excessively high early mortality could potentially obscure these processes. The lack of mortality observed enabled a more precise assessment of both structural and functional alterations in the lymphatic system throughout injury and recovery, which is essential for elucidating the therapeutic potential of enhancing lymphangiogenesis in sepsis‐associated ALI.

In recent years, multiple investigations have examined the protective properties of Ginsenoside Rg1 in sepsis‐induced ALI through diverse mechanisms, collectively enhancing our comprehension of the compound's therapeutic potential. This review juxtaposes these prior findings with our own results to underscore the complementary nature of these studies.

Bao et al. demonstrated that Rg1 mitigates LPS‐induced ALI by suppressing inflammatory responses and promoting M2 macrophage polarization [38]. Their work elucidated the immunomodulatory capacity of Rg1, revealing its ability to reduce pro‐inflammatory cytokine production and inhibit NF‐κB activation, thereby attenuating the inflammatory cascade. While this approach aligns with a conventional anti‐inflammatory paradigm, our study introduces a conceptually distinct strategy by emphasizing the promotion of resolution pathways. Specifically, we provide evidence that Rg1 facilitates functional lymphangiogenesis via the VEGFC/D‐VEGFR3 signaling axis, actively enhancing the clearance of edema and inflammatory mediators from damaged pulmonary tissue. These findings suggest that Rg1 not only suppresses inflammation but also restores physiological drainage mechanisms, thereby complementing the anti‐inflammatory effects reported by Bao et al. Moreover, considering that Rg1 promotes the polarization of macrophages toward the M2 phenotype and that M2 macrophages have been demonstrated to secrete VEGFC [29], we put forward a hypothesis that merits further exploration regarding the underlying mechanisms through which Rg1 facilitates the transition of M0 macrophages to the M2 subtype and the consequent production of VEGFC. This hypothesis delineates a definitive trajectory for subsequent research endeavors.

Liu et al. identified that Rg1 activates the PGC‐1α/Nrf2 signaling pathway by destabilizing FBXO3 in an m6A‐dependent manner, thereby enhancing mitochondrial function in lung epithelial cells [39]. This study provides critical insights into the intracellular protective mechanisms of Rg1, particularly in preserving mitochondrial integrity and mitigating oxidative stress at the cellular level. In contrast, our research focuses on an extracellular, tissue‐level mechanism—namely, the regeneration of the lymphatic vasculature. While Liu et al. elucidated how Rg1 confers intracellular protection to lung epithelial cells, we demonstrate that Rg1 orchestrates lymphatic vessel remodeling, establishing an efficient drainage network to remove pathological fluid and inflammatory mediators from the lung interstitium. These mechanisms are likely complementary: intracellular mitochondrial preservation maintains cellular viability, whereas extracellular lymphatic remodeling facilitates the effective clearance of accumulated deleterious substances.

Zhong et al. employed network pharmacology and molecular dynamics simulations to identify AKT1 as a pivotal target, showing that Rg1 attenuates alveolar epithelial cell apoptosis via the PI3K‐Akt signaling pathway [40]. Notably, our study also observed AKT activation downstream of VEGFR3 signaling following Rg1 administration. However, the functional consequences differ: Zhong et al. emphasized AKT's role in inhibiting apoptosis in alveolar epithelial cells, whereas our findings indicate that AKT activation in the context of lymphangiogenesis promotes lymphatic endothelial cell proliferation, migration, and functional maturation. This latter observation aligns with prior studies [39] that have established the critical involvement of the AKT pathway in lymphatic endothelial cell motility. Thus, the PI3K‐Akt pathway appears to mediate distinct yet complementary effects of Rg1, fostering epithelial cell survival while simultaneously driving lymphangiogenesis in lymphatic endothelial cells.

Although previous research has elucidated significant anti‐inflammatory, anti‐apoptotic, and mitochondrial protective effects of Rg1, the role of the lymphatic system in Rg1‐mediated pulmonary protection has not been addressed. To our knowledge, the present study is the first to provide evidence that, in an acute sepsis model, Rg1 promotes functional lymphangiogenesis via the VEGFC/D‐VEGFR3 axis, thereby enhancing lymphatic clearance of edema and inflammatory mediators. This “drainage‐over‐suppression” strategy represents a novel therapeutic paradigm, shifting the focus from mere inflammation suppression to active promotion of resolution. Collectively, alongside prior findings, our work suggests that Rg1 exerts multifaceted protective effects in sepsis‐induced ALI, encompassing intracellular protection (mitochondrial function, anti‐apoptosis), immunomodulation (M2 macrophage polarization, cytokine suppression), and tissue‐level clearance (lymphatic remodeling). We posit that this comprehensive understanding positions Rg1 as a promising adjunctive therapeutic agent for sepsis‐associated acute lung injury.

The present study has several limitations that warrant consideration. First, although we pharmacologically demonstrated the effect of Ginsenoside Rg1 on the VEGFC/D‐VEGFR3‐ERK signaling axis, we did not utilize lymphatic endothelial cell‐specific conditional knockout mice to provide genetic and causal validation of this pathway within our model. While the role of this axis in lymphangiogenesis is well‐established in the literature, such genetic evidence would offer more direct confirmation and help exclude the possibility that Rg1 indirectly modulates VEGFC/D secretion by influencing other cell types. Second, our investigation employed an intraperitoneal LPS injection model to induce systemic inflammation and lung injury. Although this model is characterized by excellent reproducibility and a well‐defined phenotype, its pathophysiological processes differ from those of clinically prevalent sepsis models, such as cecal ligation and puncture. Future studies incorporating multiple models would provide more comprehensive and translatable insights. Third, the present work focused exclusively on the efficacy of Ginsenoside Rg1 as a monotherapy. Given that clinical sepsis management is multifaceted and integrative, exploring the potential synergistic or additive effects of Rg1 in combination with standard therapies (e.g., antibiotics, fluid resuscitation, and corticosteroids) is essential for accurately assessing its clinical utility and constitutes a primary focus of our future research. Fourth, this study did not elucidate the precise mechanisms by which Rg1 treatment leads to increased systemic and local VEGFC/D levels, including its possible effects on platelet activation or macrophage M2 polarization. Addressing this gap through in vitro cellular experiments to clarify the underlying mechanisms represents an important direction for subsequent investigations. Finally, although changes in *CCL21a* and *ACKR2* mRNA expression were detected, limitations related to commercial antibodies and time constraints precluded validation at these expression changes at the protein level. Therefore, further studies are required to confirm the specific changes in lymphatic function related to chemokine‐mediated immunomodulation at both the protein and functional levels. Despite these limitations, the core conclusion of this study remains robust: Ginsenoside Rg1 mitigates sepsis‐induced ALI by promoting lymphangiogenesis via the VEGFC/D‐VEGFR3 axis. While these limitations underscore important avenues for future research, our findings establish Ginsenoside Rg1 and the enhancement of lymphatic clearance as a novel and promising adjunctive therapeutic strategy for sepsis‐induced ALI.

Among the limitations identified, one particular observation merits further examination: although treatment with Rg1 significantly enhanced lymphatic vessel density, the expression levels of genes associated with lymphatic function (*CCL21a* and *ACKR2*) did not completely return to baseline control levels within the 72‐h monitoring period. This phenomenon likely indicates a temporal dissociation between lymphangiogenesis and the acquisition of full functional maturity, as structural regeneration typically precedes complete functional recovery in biological systems. Nonetheless, the partial normalization of these gene expressions, alongside substantial improvements in pulmonary edema, vascular permeability, and histopathological damage, supports the inference that Rg1‐induced lymphangiogenesis plays a role in the resolution of ALI. Future investigations incorporating extended observation durations and functional assessments of lymphatic activity will be essential to comprehensively delineate the temporal progression of lymphatic functional maturation following Rg1 administration.

## 5 Conclusions

In summary, this study elucidated a previously unrecognized protective role of the natural compound Ginsenoside Rg1 in sepsis‐induced ALI. Our results demonstrate that Rg1 confers protection by activating the VEGFC/D‐VEGFR3 signaling axis and its downstream ERK and AKT pathways, thereby promoting robust and functional lymphangiogenesis. This enhancement of the lymphatic network facilitated the accelerated clearance of pathological edema and inflammatory mediators from the injured lung. Our findings not only reveal a novel mechanism of action for Ginsenoside Rg1 but also provide preclinical validation for an innovative adjunctive strategy in sepsis management. This strategy advocates a shift from solely suppressing inflammation to actively promoting resolution as a complementary approach by harnessing the body's intrinsic clearance capacity. Consequently, Ginsenoside Rg1 represents a promising and clinically translatable candidate for the adjunctive treatment of sepsis‐associated ALI.

## Funding

This work was supported by National Key Research and Development Program of China [Grant number 2024YFC3505704] and National Natural Science Foundation of China [Grant number 81773373].

## Ethics Statement

The experiments were conducted in compliance with the National Research Council's Guide for the Care and Use of Laboratory Animals (China), the Animal Research: Reporting of In Vivo Experiments (ARRIVE) guidelines, and the European Union Directive 2010/63/EU on the protection of animals used for scientific purposes. All procedures were approved by the Animal Protection and Use Committee of Beijing Friendship Hospital, Capital Medical University (Ethics Approval No.: 25‐2060).

## Conflicts of Interest

The authors declare no conflicts of interest.

## Data Availability

The data that support the findings of this study are available from the corresponding author upon reasonable request.
